# Neural activity and fundamental learning, motivated by monetary loss and reward, are intact in mild to moderate major depressive disorder

**DOI:** 10.1371/journal.pone.0201451

**Published:** 2018-08-02

**Authors:** Michael Moutoussis, Robb B. Rutledge, Gita Prabhu, Louise Hrynkiewicz, Jordan Lam, Olga-Therese Ousdal, Marc Guitart-Masip, Peter Fonagy, Raymond J. Dolan

**Affiliations:** 1 Wellcome Centre for Human Neuroimaging, University College London, London, United Kingdom; 2 Max Planck—UCL Centre for Computational Psychiatry and Ageing Research, London, United Kingdom; 3 Department of Radiology, Haukeland University Hospital, Bergen, Norway; 4 Aging Research Centre, Karolinska Institute, Stockholm, Sweden; 5 Developmental Neuroscience Unit, Anna Freud Centre, London, United Kingdom; Brain and Spine Institute (ICM), FRANCE

## Abstract

**Introduction:**

Reduced motivation is an important symptom of major depression, thought to impair recovery by reducing opportunities for rewarding experiences. We characterized motivation for monetary outcomes in depressed outpatients (N = 39, 22 female) and controls (N = 22, 11 female) in terms of their effectiveness in seeking rewards and avoiding losses. We assessed motivational function during learning of associations between stimuli and actions, as well as when learning was complete. We compared the activity within neural circuits underpinning these behaviors between depressed patients and controls.

**Methods:**

We used a Go/No-Go task that assessed subjects’ abilities in learning to emit or withhold actions to obtain monetary rewards or avoid losses. We derived motivation-relevant parameters of behavior (learning rate, Pavlovian bias, and motivational influence of gains and losses). After learning, participants performed the task during functional magnetic resonance imaging (fMRI). We compared neural activation during anticipation of action emission vs. action inhibition, and for actions performed to obtain rewards compared to actions that avoid losses.

**Results:**

Depressed patients showed a similar Pavlovian bias to controls and were equivalent in terms of withholding action to gain rewards and emitting action to avoid losses, behaviors that conflict with well-described Pavlovian tendencies to approach rewards and avoid losses. Patients were not impaired in overall performance or learning and showed no abnormal neural responses, for example in bilateral midbrain or striatum. We conclude that basic mechanisms subserving motivated learning are thus intact in moderate depression.

**Implications:**

Therapeutically, the intact mechanisms identified here suggest that learning-based interventions may be particularly effective in encouraging recovery. Etiologically, our results suggest that the severe motivational deficits clinically observed in depression are likely to have complex origins, possibly related to an impairment in the representation of future states necessary for long-term planning.

## Introduction

One in 12 people suffer from major depressive disorder (MDD) at some point in their lives [1]. Yet ‘depressive disorder’ is unlikely to be a well-circumscribed entity and may be no more specific than was the ascription ‘fever disorder’ [2], where the latter can reflect the presence of different pathologies. Lack of motivation is a central feature in MDD diagnostic criteria [3], and patients report this deficit as a source of on-going personal suffering. Health professionals, patients and relatives often blame lack of motivation for the impoverished efforts of patients to improve their condition. Studying central features of MDD like amotivation may enable a better dissection of both the underlying neurobiology, information-processing and computational processes that it subserves.

It is unclear how disturbances in motivation contribute to the genesis of depression. If outcomes that make most people happy have, in some, a reduced motivational power, they are likely to be pursued less. This in turn may provide fewer opportunities for rewarding experiences. Conceivably, if depressed mood reduces motivation below a certain threshold this would lead to a vicious cycle of reduced rewards, lower mood and lower goal pursuit. This in turn could lead to the functional decompensation we call clinical depression. Both biological and psychological theories of the maintenance of depression invoke such vicious cycles. Indeed, interrupting the vicious cycle of avoiding potentially rewarding activities and subsequent low mood is an important theoretical basis for cognitive-behavioral and especially behavioral activation therapies [4,5]. These therapies are largely effective, but the mechanisms remain unclear. Detailed mechanistic testing is required [6].

One core concept informing the vicious cycle hypothesis is anhedonia, a symptom that looms large in the context of clinical depression. Anhedonia, subjectively reported as a lack of pleasure in response to ordinarily rewarding experiences, is hypothesized to reflect an endophenotype of reduced responsiveness to reward [7] predisposing to depression. This has been termed decisional anhedonia. Early studies showed that depression, and anhedonia specifically, is associated with reduced reward sensitivity more than with other aspects of reinforcement learning [8], consistent with the hypothesis of decisional anhedonia. However, for this to lead to depression through a ‘vicious cycle’, a reduced average reward rate should lead to lower mood. This has been challenged at the population level, albeit controversially. For example, the variation of the gross domestic product of developed countries bears an unexpectedly weak relation to the mood of their population, as reflected by surveys of subjective well-being [9]. It has also been challenged with respect to non-clinical individuals over short timescales [10]. Moreover, also challenging clinical stereotypes, we recently showed that receiving surprising monetary rewards (and indeed losses) has a comparable neural and emotional impact in moderately depressed patients to that seen in healthy control participants [11]. This suggests that depressed patients may behave maladaptively because of processes located further downstream in the processing of rewards and losses, making it imperative to study how patients learn and motivate action on the basis of experienced rewards and losses.

Learning-dependent motivation can be powerfully characterized by assessing the propensity to pursue rewards and avoid losses via tasks that tap into neuro-computational processes. Healthy, motivated adults have a tendency to emit action in the presence of potential rewards and to withhold action in the face of potential losses. Over and above this fundamental propensity, they quickly learn whether action or inaction leads to desired outcomes. This motivational structure has been well characterized by the orthogonalized Go–No-Go task, both during learning and after performance plateaus [12]. Withholding action (‘No-Go’) is difficult to learn in a context of potential reward, while emitting action (‘Go’) is difficult to learn in a context of potential loss. In a natural environment, these Pavlovian biases usefully guide decision-making, acting as baseline, or prior, beliefs about the right decision given an expectation of reward or punishment respectively. ‘Expecting reward’ should increase the prior belief that ‘engaging with the stimulus’ is the right decision, whereas ‘expecting loss’ should favor ‘holding back’ [13]. We thus hypothesized that depression would be (a) associated with disturbed Pavlovian guidance of action selection, as well as (b) a reduced overall impact of anticipating given rewards or losses on motivated action.

If a disturbance in Pavlovian guidance of action selection contributes to the etiology of depression, we would expect blunted Pavlovian guidance of action (i.e., less bias) in this condition. Pavlovian amotivation could reduce the effective belief that ‘engage’ (‘Go’) would bring gains in contexts where rewards are common. This could then contribute to a vicious cycle as above. The situation with regards to avoiding punishment is more complex [14,15]. In mild and moderate MDD, where anxiety is prominent, the propensity towards ‘Go’ (active avoidance) in a context of potential losses might be preserved or enhanced.

Blunted ‘reward sensitivity’, or the motivational impact of rewards on behavior, is therefore likely to be reduced in depression. Here, we tested whether moderately depressed patients show reduced sensitivity to appetitive outcomes, versus an alternative, ‘intact sensitivity’ null hypothesis. We tested this in the context of learning through well-characterized computational modeling of behavior. This parallels and complements our group’s findings regarding unchanged reward prediction error processing [11] in a non-learning context. At this moderate level of illness, and in the absence of explicit threats, we expected that processing aversive events would be preserved [16] or even enhanced, consistent with the high levels of defensive avoidance reported in depression [17]. At the neural level, we hypothesized that brain areas whose activation mirrors the value of emitting actions (Substantia Nigra / Ventral Tegmental area (SN/VTA), ventral striatum (VS), medial prefrontal cortex (mPFC)) would be less active in depression in response to action-associated cues. This would reflect blunted reward sensitivity. By contrast, regions activated in response to cues associated with withholding actions (hippocampus, inferior frontal gyrus (IFG)) would not differ from controls [18]. Finally, we sought to corroborate the findings regarding preserved reward prediction errors (RPEs) in depression that we recently reported [11] in the same participants.

## Methods and materials

### Participants

We recruited working-age adults from primary medical and psychological care services. Participants were recruited from North London between November 2012 and July 2014. Depressed participants had major depressive disorder, or its ICD-10 counterpart (Table 1). Ecological validity and generalizability were maximized by requiring health impairment to be severe enough to require diagnosis and active treatment by a qualified doctor or psychologist (in the UK, milder presentations are usually managed by less qualified personnel with support, self-help). All participants gave written informed consent and did not have other major psychiatric or medical diagnoses, neurological impairments or trauma, moderate or severe learning disability, claustrophobia or left-handedness. The study was approved by the City and East London Research Ethics Committee (11/LO/0250).

**Table 1:** Key demographic and clinical characteristics of participants.

	Scanning sample N = 53		Total sample N = 61	
	Depression	Healthy	Depression	Healthy
Gender, M:F	12:21	10:10	17:22	11:11
Fisher exact p of gender difference in proportion	0.39		0.79	
Age, Mean (SD)	33.8 (8.8)		33.8 (9.0)	
	33.7 (9.1)	34 (8.25)	34.12 (9.47)	33.2 (8.25)
Education years	16.33 (2.34)	16.45 (1.88)	16.3 (2.33)	16.7 (1.96)
Hamilton Depression, Mean (SD)	17.15 (3.4)	0.85 (1.01)	16.9 (3.69)	0.85 (1.23)
Any recreational drugs	8/33	2/20	8/39	2/22
Fisher exact p of recr. drugs diff. in proportion	0.29		0.30	
Any medication	28/33	5/20	32/39	5/22
Any antidepressant	24/33	0/20	28/39	0/22

We matched healthy and depressed groups by age, gender, socioeconomic status and years of education. Data for this study were provided by 39 depressed and 22 healthy participants. MRI safety screening allowed us to scan 33/39 depressed and 20/22 healthy participants. Participants received a flat fee per hour of the study, travel reimbursement, plus their monetary winnings in the cognitive tasks. All the participants in this study also participated in a previous study [11].

### Cognitive tasks

**Fig 1:**
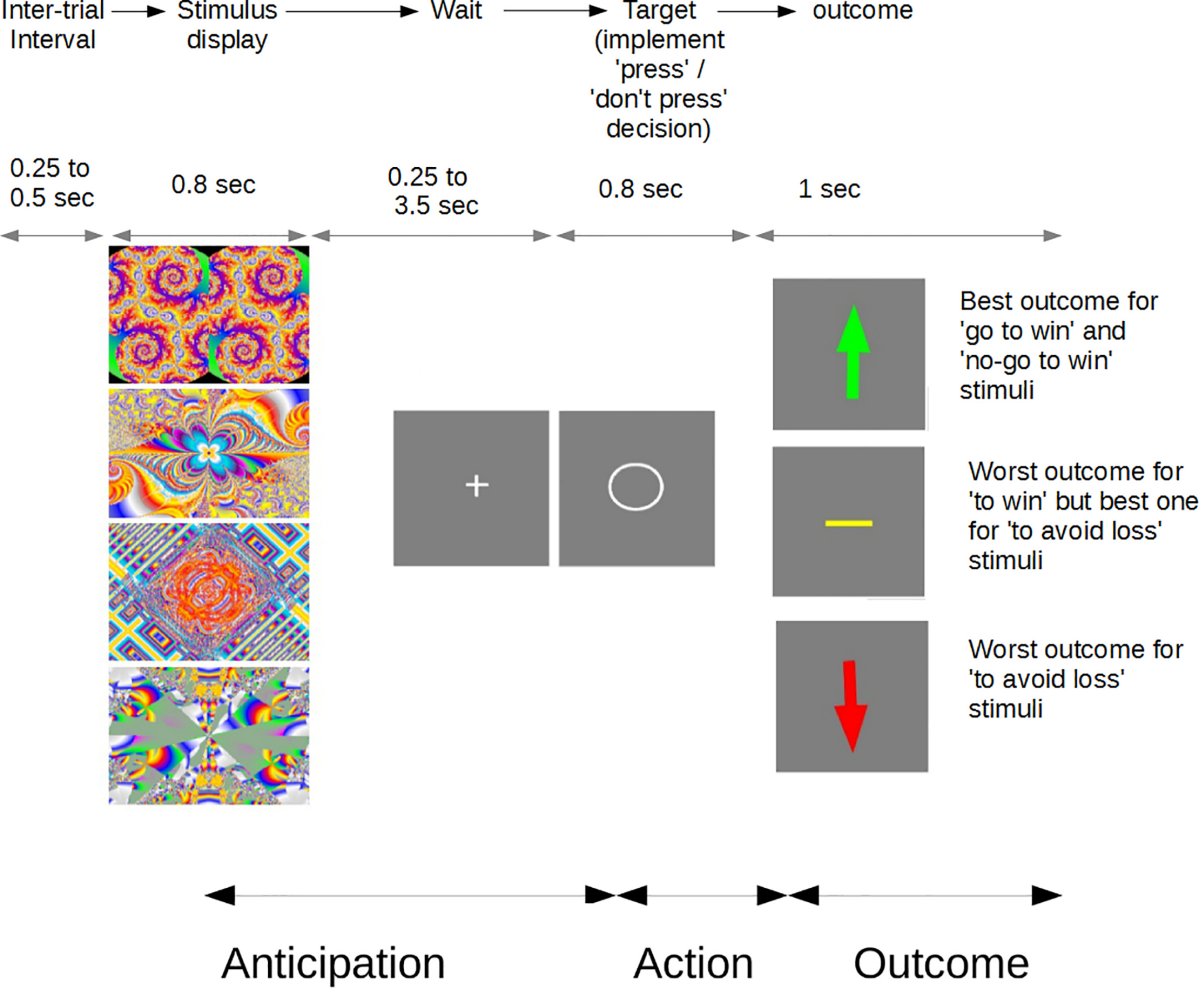
Go-No-Go task trial structure. One of four abstract stimuli was presented, followed by a waiting period (+). This, as well as the inter-trial interval, were jittered as shown to aid fMRI analysis. The participant’s decision, either to ‘Go’ or to ‘not Go’, was implemented when the target (o) appeared. The best action was followed by the best outcome 80% of the time. For example, if the second stimulus down was a ‘Go to avoid loss’ one, then quickly pressing the button when the circle appeared would result in a null outcome (yellow horizontal line) 80% of the time, and a loss outcome (downward arrow) 20% of the time. The stimuli were randomized as to their best action and outcome across participants. Before scanning, in the ‘discovery’ version of the task, suboptimal action would attract 20% best outcomes, but during scanning suboptimal action never led to the best outcome. No deception was involved at any point.

Just before the scanning session, participants performed a Go/No-Go task involving learning [12]. The task was to discover, by trial and error, whether each of four abstract stimuli required pressing a button (Go) or holding back on pressing (No-Go; see Fig 1). It was explained that during this phase of the task the correct action would be followed by the ‘best outcome’ 80% of the time and the incorrect action by the ‘worst outcome’ 80% of the time. Participants had to find out which ‘best’ and ‘worst’ outcome and action were for each stimulus. For some stimuli the best was ‘win’ and worst was ‘nothing’ (null), while for some best was ‘nothing’ and worst was ‘lose’. A stimulus could hence belong to one of four conditions: Go-to-Win (GtW), Go-to-Avoid-Loss (GtAL), NoGo-to-Win (NGtW) and NoGo-to-Avoid-Loss (NGtAL). We stressed to the participants the probabilistic nature of the task and instructed them to apply a trial and error strategy, especially if the correct responses were not clear.

Each trial started with presentation of a fractal image followed by a fixation cross and thereafter a target. Only then did participants implement their decision (Go or No-Go). ‘Go’ actions always involved the same button and had to be emitted within 700 ms after target appearance. Once participants completed the ‘discovery’ task (144 trials), they were explicitly trained on the correct responses. They were instructed on the correct answers and practiced responding until they attained over 90% performance in the exact task that they subsequently performed in the scanner (see S1 File). Despite older age than subjects in earlier studies with this task, and a potential for psychomotor retardation, depressed participants achieved in-scanner success rates equivalent to the healthy controls [19]. This was important in order to avoid performance-related confounds. We emphasized that exactly the same responses would be the correct ones for the scanning part of the experiment, where seeming failures should be attributed to chance and not to changes in action-outcome contingencies. Participants then performed the ‘trained’ version of the task in the scanner, following the above description and as per the published paradigm [20]. The task consisted of two 12-min blocks. Each ‘win’ was worth £0.40, making average performance-related fees about £15 overall.

### Computational modeling

Behavior in the ‘discovery’ phase was fitted with computational models based on published work [12,13,20]. The core model that we used was the one shown to perform best in the literature. All models we used comprised of two parts. First, at each trial a Rescorla-Wagner or Q-learning like rule [21] updated the values of the presented stimuli (*V*) and values of the actions taken (*Q*) according to a constant learning rate *λ* and a prediction error. The latter was an estimate of the extent to which expectations about reward were violated (Eqs 1 and 2 below). In this first part, the value of actions was calculated in an unbiased form. The core model contained two different return sensitivities (aka motivational exchange rates, or inverse temperatures) *ρ*_*v*_ depending on whether a positive or negative return *r* was received. In our case, *r* could take the values of +1 (win), -1 (loss) or 0 (null outcome):

$$Q_{t+1}=Q_{t}(a_{t},s_{t})+\lambda (\rho_{v}r_{t}-Q_{t}(a_{t},s_{t}))$$ Eq 1

Only *Q* values pertaining to realized stimuli and actions were updated–all others were carried forward from the previous trial. All models also kept track of the state values pertaining to each stimulus using the same parameters:

$$V_{t+1}=V_{t}(s_{t})+\lambda (\rho_{v}r_{t}-V_{t}(s_{t}))$$ Eq 2

Crucially, in the second part of the models the *Q* values were biased by up to two terms. Both were included in the core model, and represented an overall tendency towards action (‘Go bias’) and a Pavlovian bias that depended on valence (state value):

$$q_{t}(a_{t},s_{t})=Q_{t}(a_{t},s_{t})+b_{go}(a_{t})+b_{pav}(a_{t},V_{t}(s_{t}))V_{t}(s_{t})$$ Eq 3

Both the bias coefficients *b* assume zero values unless *V_t_*(*s_t_*)>0 (for both) and *a_t_* = *Go* ≔ 1 (for *b_pav_*). This means that the NoGo action and aversive context are taken as reference. Finally, the policy probability for choosing an action in all models was given by the softmax function, albeit squashed by a lapse rate parameter *ξ*:

$$p(a_{t}|s_{t})=(1-\xi )\frac{\exp(q(a_{t},s_{t}))}{\sum_{k}(exp(q(a_{k},s_{t}))}+\frac{\xi }{2}$$ Eq 4

The core model thus had six parameters, appetitive and aversive sensitivity, learning rate, action bias, Pavlovian bias and lapse rate. We compared this against alternatives that did not contain biases and/or multiple motivational exchange rates.

Within the core model, the motivational exchange parameters and learning rate operationalized our hypotheses that depressed participants would show reduced sensitivity to reward (reduced motivational exchange for rewards). The second part of the model was not concerned with learning, but with making the decisions to emit or withhold actions. It boosted action-values for ‘Go’ by a constant Go-bias parameter but also a Pavlovian term proportional to the value of the stimulus in question. Thus ‘Go’ was boosted for appetitive stimuli and penalized for aversive stimuli (Eqs 3 and 4). The coefficient of the Pavlovian term allowed us to operationalize the hypothesis that Pavlovian bias would be aberrant in depression.

In order to test for differences between the two groups, we fitted behavior with maximum-a-posteriori (MAP) statistical models, providing minimal regularization of the fitted parameters, as using empirical priors during Expectation-Maximization EM fitting [20,22] risks suppressing true extreme values [23] and thus biasing group comparisons. The MAP approach is also suitable to compare groups on an equal footing with respect to the parameters quantifying our hypotheses, so that the Pavlovian bias and motivational exchange rate parameter estimates would be available for both groups by construction. We also performed EM analyses to test whether patients and controls may have used different cognitive models, as has been found with other patient groups [22].

### Psychiatric measures

Hamilton Depression Rating scale (HDRS-17) scores were our primary measure of depression severity [24]. This was used in correlational analyses. We also administered the Patient Health Questionnaire– 9 (PHQ-9), the Beck Depression Inventory–II (BDI-II) and the Snaith-Hamilton Pleasure scale (SHAPS). All participants provided HDRS-17 and PHQ-9 data. 14 healthy and 24 clinical participants provided BDI-II and SHAPS data.

### Imaging

We used a 3-Tesla Trio scanner (Siemens, Erlangen, Germany) with a 32 channel head coil. Whole-brain T2*-weighted echo-planar imaging (EPI) data were acquired using a sequence designed to minimize dropout in the striatum, frontal cortex, and amygdala [25]. Each volume contained 43 slices of 3-mm isotropic data (echo time = 30 ms, repetition time = 3.01 s, slice tilt of -30 degrees, Z-shim of -0.4 mT/m ms, ascending slice acquisition order. To account for T1 saturation effects, the first 5 volumes of each scan were discarded. Field maps were acquired to allow for subsequent geometric distortion correction and T1-weighted images were acquired for structural alignment. Physiological monitoring included measurements of pulse and breathing using the Spike2 data acquisition system (Cambridge Electronic Design Limited, Cambridge, UK). Preprocessing of the EPI data followed standard procedures [26], i.e. EPI unwarping using field maps, slice-time correction to the first volume, motion correction, spatial transformation to the Montreal Neurological Institute (MNI) template, spatial smoothing with a Gaussian kernel of 8-mm full-width at half-maximum. Analyses used the Statistical Parametric Mapping package (SPM; www.fil.ion.ucl.ac.uk/spm).

Functional analyses were also performed in SPM (version 12b; www.fil.ion.ucl.ac.uk/spm). In order to assess brain activity during decision-making, i.e. in anticipation of action, we first formed regressors by convolving a canonical hemodynamic response with stick functions at the onset of the fractal images for each of our four conditions of interest (Go-to-win, Go-to-avoid-loss etc.) for each participant. The analyses included a regressor devoted to decisions which were followed by a motor response, so as to parcel out variance associated with actual motor execution. All models also included three regressors describing rigid-body translational and three describing rotational movement of the head, resulting from the realignment analysis during pre-processing, to reduce movement artifacts.

We created regressors of interest for outcome-onsets, separately for win, loss and null outcomes. Here only correct response trials were included. These were complemented by separate regressors of no interest, which modelled trials where the stimulus was not followed by a correct response. Incorrect responses were few (<5%) and likely to reflect processes, such as attentional lapses, underpinned by mechanisms unrelated to the study goals. We then performed hypothesis testing regarding differences in contrasts of interest with respect to psychopathology in three ways. First, we used functional regions of interest (ROIs) based upon areas showing task related activation or de-activation in all participants combined. We performed a 2x2 ANOVA with factors Go/No-Go and win/lose and looked across the entire sample for clusters significant at 5% FWE for the contrasts in question, but *not* for between-group differences, thereby avoiding ‘double-dipping’ [27]. For example, the ‘greater activation for action emission’ contrast was (Go-to-win + Go-to-Avoid-Loss)–(No-Go-to-Win + No-Go-to-Avoid-Loss). These significant clusters defined the functional ROIs. Second, we used predefined ROIs, i.e. clusters of significant contrast within specific anatomical areas (e.g. SN/VTA) where activity was found to vary with choice in previous work [20]. In both cases we formed contrasts using averaged ROI activations and compared them between groups. Third, we formed regressors corresponding to contrasts of interest at the first (individual) level and performed whole-brain, between-group t-tests at the group level (also see S1 File). For exploratory analyses, we first used more liberally thresholded but similarly formed ROIs. Finally we explored brain responses to reward prediction errors as per our previous analyses [28]. Thus we formed 2x2 ANOVAs, but with factors Go/No-Go vs. better-than-expected / worse-than-expected, and implemented the functional ROI-based approach above. All exploratory analyses were performed both with 5%-FWE and with 0.001-uncorrected thresholds (also see S1 File).

## Results

### Psychometric

**Fig 2:**
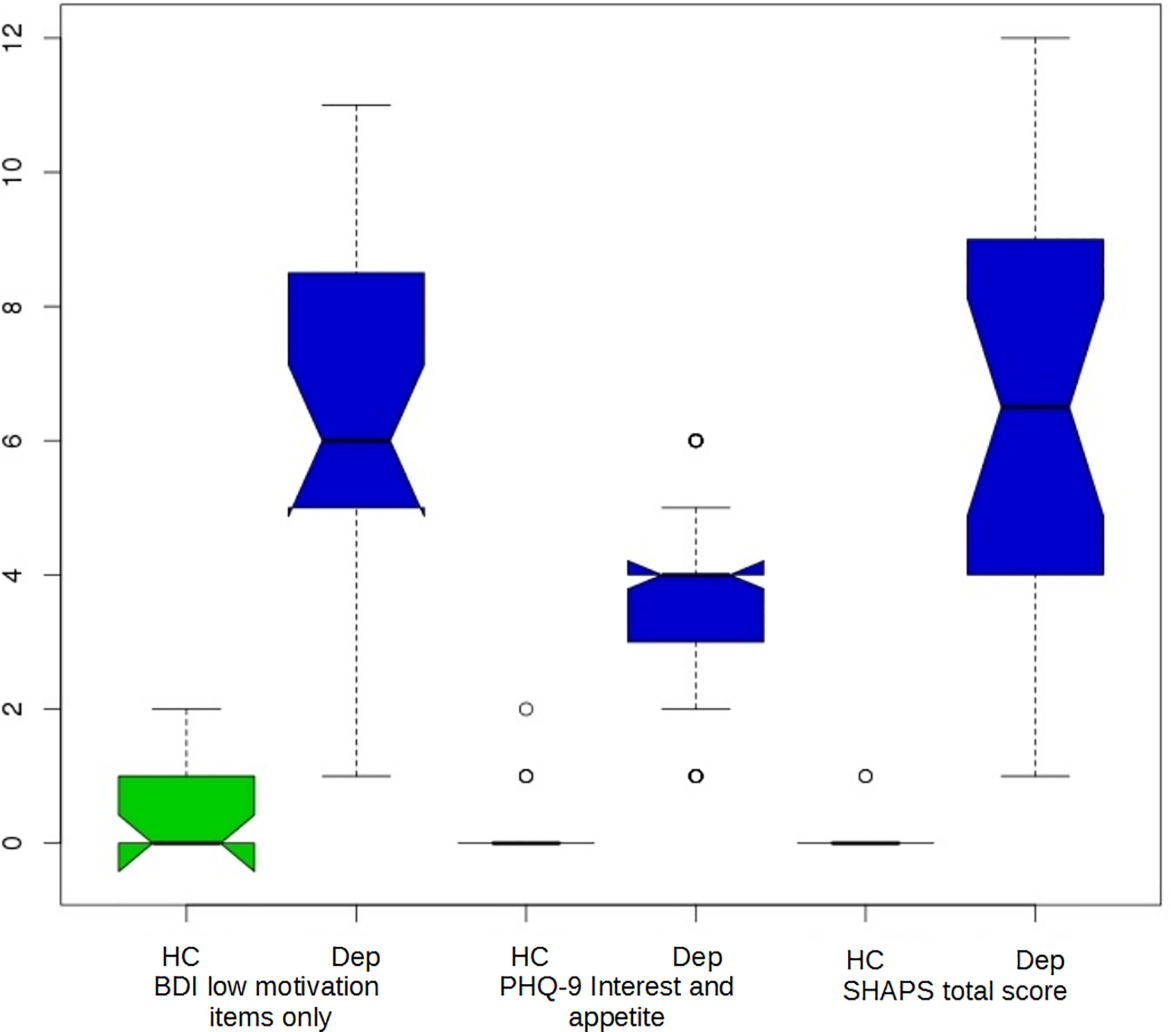
All clinical measures of motivation clearly separate the healthy control (HC, odd plots) from the depression (Dep, even plots) sample. Shown are summed items ‘loss of interest’, ‘change in appetite’ and ‘loss of interest in sex’ from the Beck Depression Inventory BDI), ‘interest’ and ‘appetite’ items from the Patient Health Questionnaire– 9 (PHQ-9) and the total score of the Snaith-Hamilton Pleasure Scale (SHAPS). For each pairwise comparison, Wilcoxon p < 1e-06.

All healthy controls scored 0–4 in the HDRS-17. 27/39 of the depression participants scored above a conventional cutoff of 14 for moderate depression, whereas using the classification of Zimmerman and co-workers, 21 scored in the ‘mild’ range (8–16), 17 in ‘moderate’ (17–23) and one in ‘severe’, i.e. over 23 [29]. We confirmed that the depression sample experienced substantial problems with the subjective experience of pleasure and interest in activities. We used the SHAPS total score and a priori clinically relevant questions from the PHQ-9 and SHAPS. No matter which measure is used, it clearly separated the depression from the healthy samples with virtually no overlap. Fig 2 specifically illustrates psychometric measures relevant to motivation.

### Behavioral

**Fig 3:**
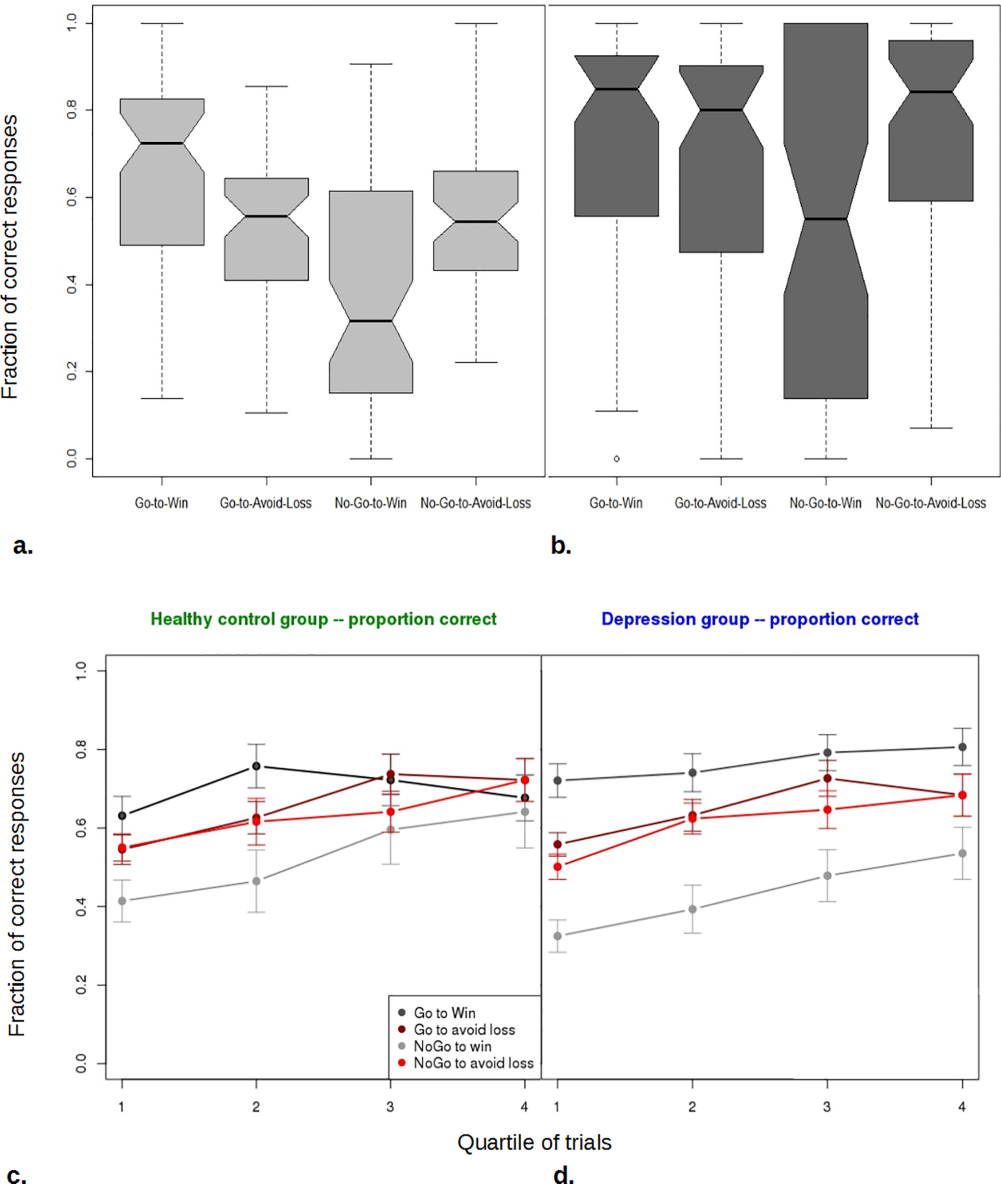
Behavior in the ‘discovery’ version of the task closely replicates previous results regarding the effect of trial valence on the propensity to act. **a.** Here, depressed and healthy groups are combined. Shaded bars: interquartile ranges; Notch: standard error of the median. During early trials participants perform much better in Go-to-Win than No-Go-to-Win, where they do worse than chance. **b.** Late trials, but otherwise as in a. All conditions show increased performance–each median has improved. However different abilities of participants to learn the optimal behavior results in greater spread of performance. This is particularly striking for the No-Go-to-Win condition. **c.** Performance in each quartile of trials for the Healthy Control group, showing learning across trials. Bars are +/- 1 standard error of the mean. **d.** As in c. but for the Depression group. Differences group differences between c. and d. are not significant.

The ‘discovery’ phase of the experiment replicated previous findings (Fig 3A and 3B). Both healthy and depressed participants performed worse when required to withhold action, compared to emit action, in order to win. This, as well as the opposite effect seen in the avoid-loss conditions are the signature of Pavlovian bias [13].

We then fitted the behavior with the ‘core model’ using uninformative priors and MAP fitting. Against our hypotheses, we found no significant differences between the groups. Table 2 shows that for the key parameters of interest, Pavlovian bias, learning rate and especially motivational exchange rate there is evidence that the two groups do not differ.

**Table 2:** Parameter comparison for the weak-prior MAP fit of the core model.

Model parameter	Bayes Factor (Scaled JZS) > 1 is in support of no difference between Depression and Healthy groups	t value	uncorr. p
Appetitive motivational exchange rate (reward sensitivity)	3.594	0.277	0.783
Aversive motivational exchange rate (punishment sensitivity)	3.291	-0.537	0.594
Learning rate	2.983	0.742	0.462
Pavlovian Bias	2.801	-0.822	0.414
Lapse rate	1.447	-1.509	0.139
Go-bias (favouring action emission)	2.365	-1.041	0.303

**Fig 4:**
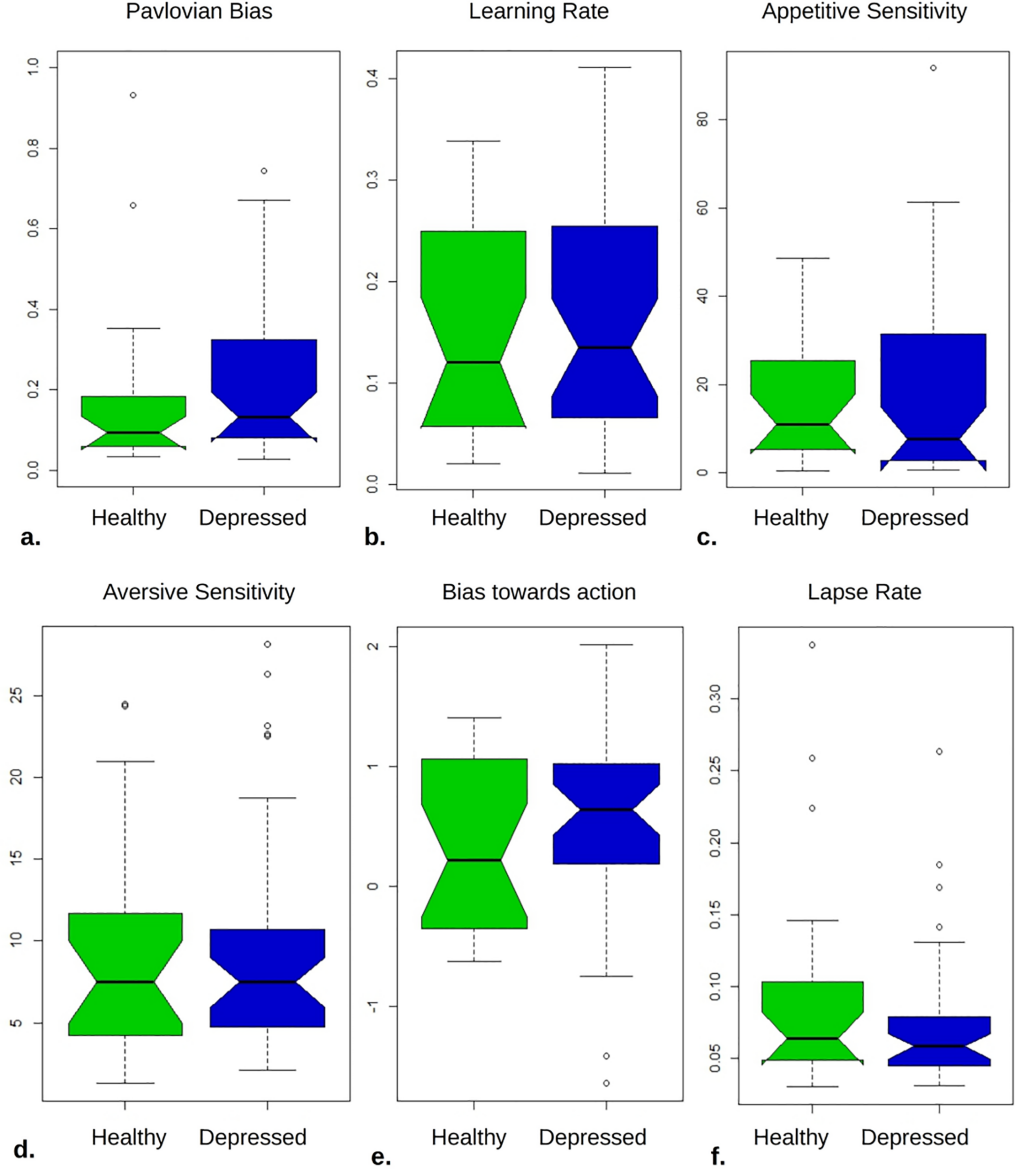
Healthy participants had similar parameter distributions to the depressed.

We then performed model comparisons (Table 3). We used the integrated Bayesian Information Criterion score derived from EM fitting to compare between models and to estimate the strength of evidence for or against using two separate sets of group-level, empirical prior distributions to estimate individual parameters. When the behaviour of the two groups was fitted separately by a variety of different models, these performed worse, in terms of integrated Bayesian Information Criterion, than the ‘core’ model, with one important exception. That is, for the healthy group the ‘core’ model with the six parameters of Table 2 came second (by 17.7 BIC units) after a model that excluded the Pavlovian bias. However, when the common empirical priors (last column) vs. separate priors comparison is taken into account, we see that overall the evidence powerfully favours the core model and common priors (by 60.83 units). Fig 4 shows visually how similar the parameter posterior distributions are for the two groups under this winning 6-parameter model.

**Table 3:** Model comparison based on the Bayesian Information Criterion and random effects EM estimation. Key: ‘b’ return sensitivity, so that ‘bb’ refers to a model with separate appetitive and aversive sensitivity; ‘a’ learning rate; ‘p’ Pavlovian bias; ‘l’ lapse rate; ‘g’ Go-bias (favoring action over inaction). Separate iBIC for the two groups, and also the total sample, are shown in columns. Using a single set of parameters to describe the group distributions for the healthy and control groups achieves a better score, by about 61 BIC units, than summing the best of each separate fit. Were one to fit the two groups separately, one would run a danger of over-fitting, here over-emphasizing differences between the groups.

	Healthy control (separately)	Depression group (separately)	Common group-level parameters
Core model (bbaplg)	3623.96	**5866.12**	**9411.59**
Core model minus go-bias (bbapl)	3690.58	6188.85	9808.36
Core model minus Pavlovian bias (bbalg)	**3606.30**	5912.36	9443.71
bbal	3674.52	6224.73	9849.59
bapl	3792.21	6399.70	10143.98
baplg	3688.10	5977.05	9594.96
balg	3676.90	6092.47	9706.65
bal	3773.06	6499.39	10232.58

The ‘trained’ version of the task used in the scanner was designed to induce equivalent performance in the two groups, guarding against differences that might be engendered by differential performance or learning. Indeed there were no performance differences, the median fraction of correct responses being 0.94 for both healthy and depression groups. The effect of trial valence on reaction time was significant, with the Go-to-win trials being faster (overall sample Wilcoxon *p* = 0.0009) but this Pavlovian effect was the same in healthy (mean RT difference = 14.8 ms) vs. depressed participants (RT diff. = 14.4 ms, *p* = 0.95 between groups). The same measure is not as valid a measure of Pavlovian bias during learning because during learning RT also reflects deliberation of which is the optimal choice. Still, we checked for differences in bias-related speeding between the groups in the discovery phase and found none.

### Imaging

**Fig 5:**
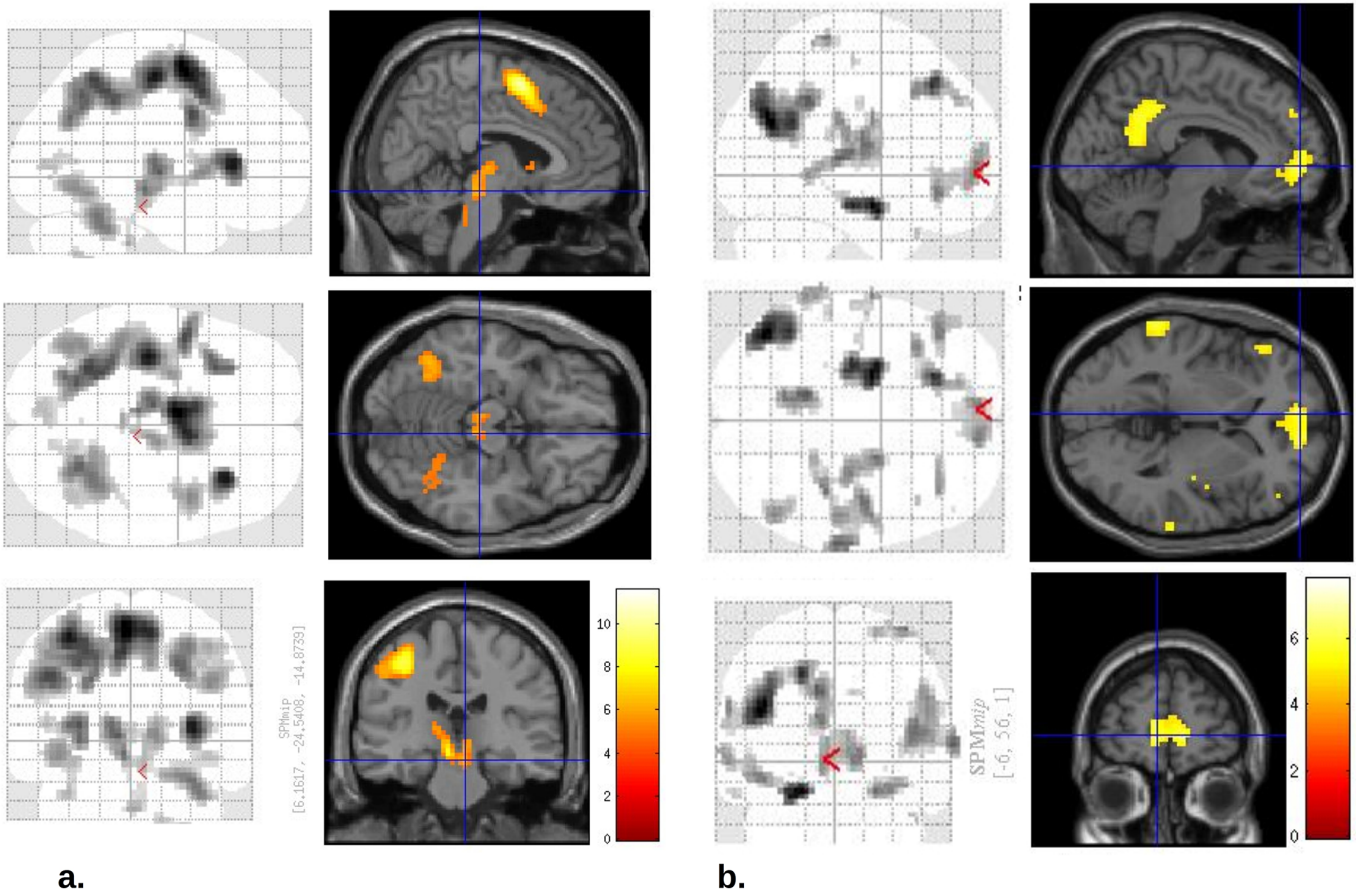
Significant activations for key contrasts, on which region-of-interest analysis was based, replicated previous findings. **a.** Go > No-Go contrast showed significant bilateral midbrain (crosshairs), caudate, insula, primary motor and supplementary motor clusters (FWE p<0.001). **b.** No-Go > Go contrast, i.e. areas activated when action was to be withheld, showed highly significant bilateral inferior frontal, medial prefrontal, posterior cingulate and hippocampal clusters, as well as a prominent left parieto-occipital cluster (all FWE p<0.001).

We replicated the main task findings from the literature in the combined sample. Fig 5 shows key clusters responding to action emission (5a) or its withholding (5b). There was no significant cluster responding to valence or to action-valence interaction that might index Pavlovian bias. We also found significant responses both for the main effect of reward prediction error (RPE) and for action x RPE interactions, with evidence for stronger RPE signals for Go actions (Figures A and B in S1 File). Better-minus-worse than expected outcome (bte-wte) contrasts showed prominent clusters in left inferior medial prefrontal cortex. Worse-better than expected outcome (wte-bte) contrasts were prominent in bilateral anterior insula. These regions also showed FWE corrected clusters at 5% significance for the respective interactions with action.

**Fig 6:**
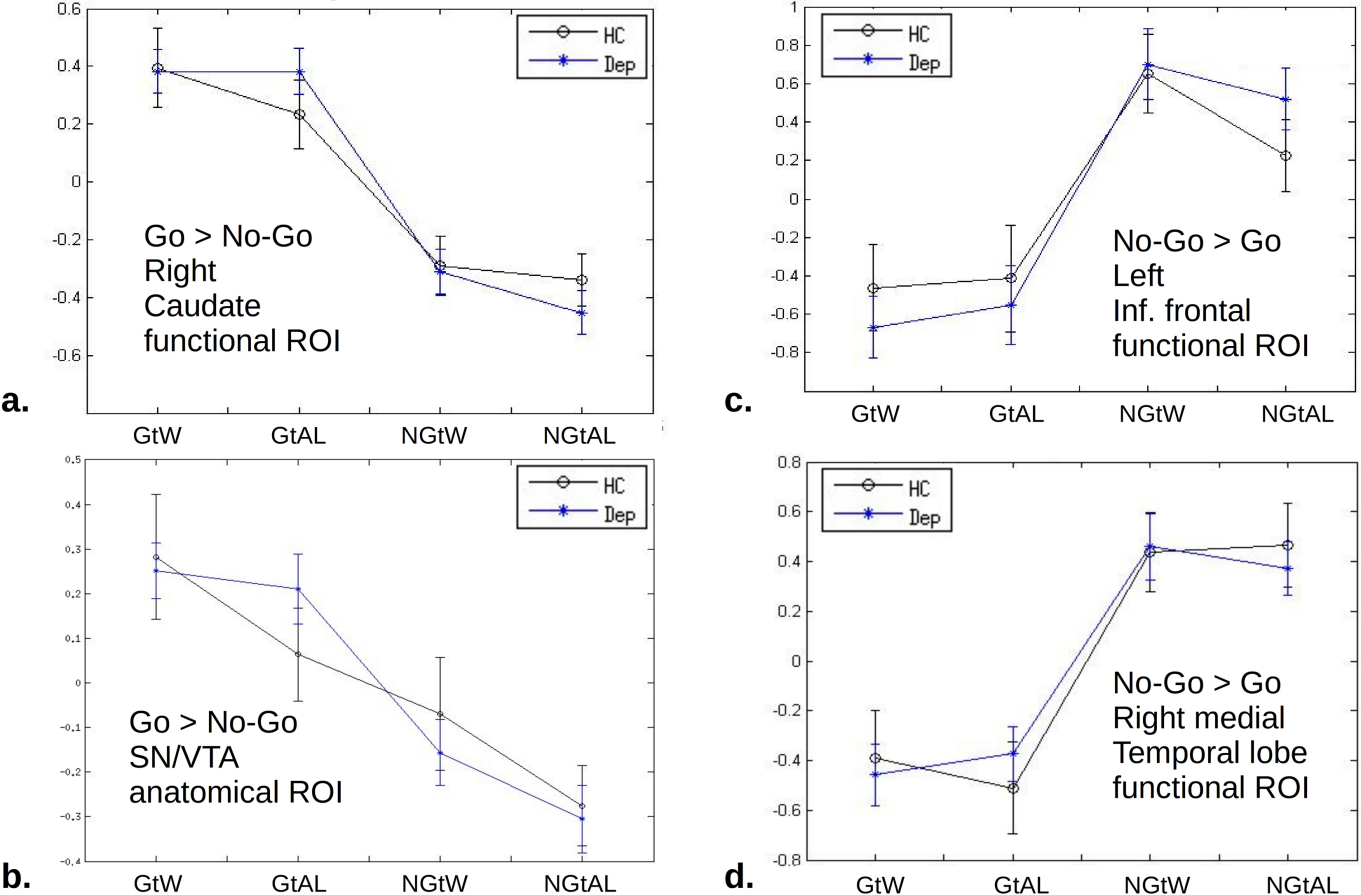
Comparisons of Healthy vs. Depression activations in specific ROIs hypothesized to be important for motivated action. Means and standard errors are shown for each of the four sets of trials. No area showed differences between depression (blue) and control (black). **a.** Right Caudate region of interest defined as the cluster significant at 5% FWE for the main effect of action over the entire sample. The ‘Go’ conditions have greater activation by construction, but no other differences are seen. **b.** As an example, this ROI is an anatomically predefined mask for Substantia Nigra / Ventral Tegmental Area. It is a subset of the cluster indicated by cross-hairs in Fig 4A. The win–avoid-loss difference is not statistically significant. **c.** Inferior frontal ROI defined by functional criteria (as per a.) of greater activation for holding back (‘No-Go’). **d.** As per (c.) but for amygdala/hippocampal cluster.

None of the contrasts that we tested, either at action anticipation or at outcome, showed evidence of dependence on depression in the hypothesis-driven analyses described above (Fig 6). As an important example, we tested for group differences with respect to the simple contrasts win-loss and loss-win, using functional regions of interest (see S1 File for more details about regressors and illustration of the functional ROIs, Figure A in S1 File). These contrasts provide evidence for positive and negative reward prediction errors, but they are not balanced for expected value in our task. Therefore the validity of this contrast as a measure of RPE relies on there being no influence of expected value on brain activation in our paradigm. Several studies [12,13,20] including the present one, have found no such main effect based on the cue-onset regressors justifying the use of this contrast. Using both 5% FWE and uncorrected <0.001 clusters, we found no significant differences between the groups.

Using HDRS-17 depression scores as a continuous measure did not yield any significant correlations with neither neural activations nor behavioral measures. In the exploratory analyses, based on functional ROIs, an activation cluster comprising bilateral supplementary motor areas showed somewhat activation in the depression group (p = 0.034 uncorrected). Failure to reject the null hypothesis of no-difference in most ROIs does not by itself constitute positive evidence for this null hypothesis. We therefore calculated Bayes’ factors (scaled Jeffrey-Zellner-Siow priors; [30]) and found that each ROI except bilateral SMA contributed moderate evidence that the null hypothesis was true. By this Bayesian criterion, the bilateral SMA showed weak evidence for the depression group showing increased activation. This is further described in ‘Exploratory findings in Supplementary Motor Area’ in S1 File.

## Discussion

We used a well-characterized paradigm and a carefully selected and matched sample of outpatients to investigate motivational abnormalities in mild to moderate but clinically important MDD. Our tasks first assessed learning, just before scanning, and then neural activation during action anticipation and during responses to outcomes. A considerable body of research [8,31,32] led us to expect that depressed participants would show blunted behavioral and neural responses to potential reward, but preserved responses in the context of potential losses. Instead, we found no such behavioral blunting and no differences in neural responses across multiple brain areas, where we hypothesized that the value of actions would be represented. In exploratory analyses we found some evidence that the bilateral supplementary motor area was slightly more activated during anticipation of action in people with depression.

This evidence argues against a hypothesis that a disturbed Pavlovian bias plays an important role in depression. Our depressed participants were carefully chosen to be representative of patients seeking and receiving active, usually pharmacologic, treatment for this condition. We can therefore say with some confidence that disturbed Pavlovian bias is unlikely to impair recovery of such patients. The situation may be quite different in severely depressed patients, where disturbed Pavlovian and learning mechanisms may play more important roles. It also remains to be seen whether the treatment that our participants received may itself have normalized their decision-making parameters. However, as brief treatment is unlikely to have modified relevant traits, our results suggest that disturbances in such parameters do not constitute important trait-like risk factors for outpatient depression.

We did not replicate a widely reported blunted response to reward in depression [33–37]. Instead, the results of this study parallel the findings of intact prediction-error processing and emotional responsivity in the same sample of depressed patients, under a different paradigm that tested for full expression of an outcome prediction error [11]. Importantly, here we distinguish between reward-dependent learning versus reward-dependent performance, as well as limiting our sample to moderate MDD. Crucially, subjects were thoroughly trained at the time of neuroimaging and so performance was equal between groups, obviating performance confounds. This performance feature might explain the intact reward responses we observed in depression. It would also imply that effects seen in previous studies may be related to decrements in performance and associated processes. Thorough training may modify how feedback is perceived from a subjective point of view, how attention may differ in depressed patients with respect to the valence of outcomes and result in rumination, etc. These processes may also impair the effective motivation of patients in ecological, non-laboratory contexts. Tentatively, we may associate such processes with reduced confidence but further studies are needed to distil them. Our study had a total N = 61 and so the absence of any trend effects even where negative findings are reported is striking. Heterogeneity of the mechanisms behind motivational disturbance in MDD may also contribute to non-replication, though the nature of this putative heterogeneity accounting for the pattern of results we observe is unclear. Furthermore, our study addressed in detail the motivating power of different types of gain and loss rather than the evaluation of different outcomes.

Our study suggests that the basic neurobiological machinery of motivated action emission and inhibition is largely preserved in moderate MDD, consistent with studies that have found other aspects of reinforcement learning to be, slightly unexpectedly, preserved or even improved in this disorder [19,38,39]. The paradigm that we used powerfully differentiated between neural activation patterns during different types of decision-making (Figs 5 and 6), so that if these patterns are disturbed in depression, then this effect is very small. Bayesian metrics (Tables 2 and 4) showed that our study provided moderately strong support in favor of the null hypothesis, going beyond the no-evidence-for-difference afforded by p-value testing [30]. Consistent with the results we present here, our group also found intact RPEs in a non-learning context [11] in the same patient sample.

**Table 4:** In regions of a priori interest, as well as in functional ROIs, evidence was found that the same activation took place in depression and control groups. The only exception was the bilateral supplementary motor area, where there was evidence for greater activation in the depression group upon action emission as contrasted with action withholding.

Key Regions of Interest	Bayes Factor (Scaled JZS)	t value	uncorr. p
Action Emission > Action Withholding during anticipation (functional ROIs)	> 1 is in support of no difference between Depression and Healthy groups		
Midbrain	1.59	-1.40	0.17
Supplementary Motor Area (bilat.)	**0.53**	**-2.17**	**0.034**
Left Caudate	3.50	-0.15	0.88
Right Caudate	3.27	-0.43	0.67
ROIs of interest a priori (structural)			
SN/VTA	1.48	-1.46	0.15
Ventral Striatum	3.03	-0.61	0.54
Action Withholding > Action Emission during anticipation (functional ROIs)			
Left Parieto-Occipital	3.52	0.078	0.94
Left Amygdalo-Hippocampal	3.41	-0.29	0.77
Right Amygdalo-Hippocampal	3.31	-0.39	0.69
ROIs of interest a priori (structural)			
SN/VTA	3.46	0.21	0.82
Ventral Striatum	3.48	0.18	0.85
Better > Worse than expected reward prediction error (funct. ROI)			
ventromedial PFC	3.47	0.20	0.84
Worse > Better than expected reward prediction error (funct. ROI)			
right insula	2.63	0.85	0.40
left insula	3.47	0.20	0.84

Both the task data and the neural data stand in contrast with the clinical data, including the subjective state of anhedonia that is arguably the self-reported counterpart of decisional anhedonia (Fig 1). This may be due to the specific learning and decision-making involved in tasks involving simple associations and small monetary gains and losses. Such tasks can be carried out well based on associative operant learning and habit-based decision-making, just as described by our mathematical model. These basic mechanisms could be largely intact, suggesting that successful psychological therapies may recruit intact learning mechanisms to destabilize separate pathogenic mechanisms, rather than targeting generalized deficits in reinforcement learning [40]. Intriguingly, a recent study found that in depressed patients, somewhat younger than in our study, those with stronger Pavlovian biases had a better prognosis [41]. Such findings point to a possible translational potential not only of pinpointing disturbances that can be targeted by treatments, but also of identifying the intact resources that can be used in rehabilitative treatments.

One alternative, phenomenologically plausible hypothesis regarding the role of motivation in depression would be that the clinically observed deficits concern the emotional component of explicitly represented, planfully achieved, future states. This would argue for a distortion in affective forecasting [42] in depression. In our task, decisional consequences of distortions of affective forecasting may be masked both by the dominance of associative learning and reliance on avoiding the worse of the two outcomes for each stimulus. Another alternative is that motivation in depression assumes pathogenic importance [16] in domains ecologically relevant to the aberrant, real-life beliefs about negative outcomes that depressed patients harbor.

Future research should target specific ways in which modestly dysfunctional reinforcement learning mechanisms may operate as part of networks of mechanistic factors, such as model-based reasoning applied to personal contingencies and the computational role of emotion [6] including affective forecasting [42]. Most importantly, rather than addressing ‘depression’ (like fever!) in general, studies of learning-based motivation should specifically investigate that minority of patients that fail to benefit from learning-based interventions, especially cognitive-behavioral therapies, which tacitly assume that patients have adequate neurobiological resources in their disposition to learn new, adaptive behaviors in therapy. Our intriguing exploratory finding (somewhat increased supplementary motor activation during anticipation of action) awaits further research. Given that it was not accompanied by changes in reaction time or neural activation during performance of action and receipt of outcome (Figure C in S1 File), if confirmed it may be related to increased subjective perception of effort in this condition [43]

The key limitation of the current study is its modest sample size, which meant that small differences between groups may have been missed. The Bayesian analysis and our companion study [11] render this less likely.

### Conclusion

Neural activation patterns subserving motivated *action* are largely intact in people with moderate depression. Other influences on learning, especially goal-directed mechanisms including affective forecasting, may also be a fruitful research avenue of investigation in depression.

## Supporting information

S1 File Supplement. Key Supplementary Material file, including additional methodological details, details of the group comparison methodology, details of analysis of brain responses to reward prediction error and exploratory findings in the Supplmentary Motor Area. It also includes Figure A, Figure B and Figure C and References to the Supplementary material. **Figure A in S1 File. Outcome contrast maps for correct trials at uncorrected threshold p = 0.001**. **a.** Win-lose. Cross-hairs at left ventral striatum. This cluster does not survive correction at 5% FWE. **b.** Lose-Win contrast. Cross-hairs at the level of pretectum. **Figure B in S1 File. Go responses (left side of each panel) elicit stronger contrasts than No-Go both in the mPFC area significantly sensitive to better-than-expected RPEs** (**a.**) and in the insular area more sensitive to worse-than-expected RPEs (**b.**). Non-overlapping notches denote significance using a conservative non-parametric (Wilcoxon) test for illustration. **Figure C in S1 File. Supplementary motor area differentially activated in the Depression vs. Healthy control group.** (**a.**) The cluster showing clearly significant greater activation during action anticipation, *p*_FWE_ < 0.01 (**b.**). Activations for the four conditions, demonstrating lesser responsiveness for the healthy group. (**c.**). Contrasts during anticipation (according to Action, i.e. anticipating Go > NoGo, according to valence, i.e. anticipating Win > Avoid Loss, interaction of the two, i.e. measure of Pavlovian bias), at the onset of action itself (the Key press) and at receipt of outcome (Win > Lose only, null outcomes excluded).
(DOCX)

S2 File Key to data and notes. This is a document detailing the variable names in the processed and raw data provided, and also how to access the imaging data uploaded into Neurovault. This document also explains the contents of the zip files HealthyControlBehavioralData.zip and DepressionGroupBehavioralData.zip which include the raw ‘discovery task’ data.
(DOCX)

S3 File Dataset—ModelParameters. A comma-separated variable spreadsheet containing the detailed model parameters derived from the ‘winning’ model of Table 3 in the main text.
(CSV)

S4 File Dataset—QuestionnaireData. Raw data for the psychometric scales used in this work.
(CSV)

S5 File Dataset–healthy control behavioral task. behavioural data from the ‘discovery’ task for the Healthy Control group.
(ZIP)

S6 File Dataset–depression group behavioral task. The behavioral data from the ‘discovery’ task for the Depression group.
(ZIP)
