# Supplementary material for: Neural activity and fundamental learning, motivated by monetary loss and reward, are intact in mild to moderate major depressive disorder
Source: PLoS One. 2018 Aug 2;13(8):e0201451. doi: 10.1371/journal.pone.0201451 (PMC6072018; doi:10.1371/journal.pone.0201451)
Supplement: S1 File — Key Supplementary Material file, including additional methodological details, details of the group comparison methodology, details of analysis of brain responses to reward prediction error and exploratory findings in the Supplmentary Motor Area. It also includes Figure A, Figure B and Figure C and References to the Supplementary material. Figure A in S1 File. Outcome contrast maps for correct trials at uncorrected threshold p = 0.001. a. Win-lose. Cross-hairs at left ventral striatum. This cluster does not survive correction at 5% FWE. b. Lose-Win contrast. Cross-hairs at the level of pretectum. Figure B in S1 File. Go responses (left side of each panel) elicit stronger contrasts than No-Go both in the mPFC area significantly sensitive to better-than-expected RPEs (a.) and in the insular area more sensitive to worse-than-expected RPEs (b.). Non-overlapping notches denote significance using a conservative non-parametric (Wilcoxon) test for illustration. Figure C in S1 File. Supplementary motor area differentially activated in the Depression vs. Healthy control group. (a.) The cluster showing clearly significant greater activation during action anticipation, p FWE < 0.01 (b.). Activations for the four conditions, demonstrating lesser responsiveness for the healthy group. (c.). Contrasts during anticipation (according to Action, i.e. anticipating Go > NoGo, according to valence, i.e. anticipating Win > Avoid Loss, interaction of the two, i.e. measure of Pavlovian bias), at the onset of action itself (the Key press) and at receipt of outcome (Win > Lose only, null outcomes excluded). (DOCX) [file pone.0201451.s001.docx]

**Supplementary material for**

**Neural activity and fundamental learning motivated by monetary loss and reward are intact in mild to moderate major depressive disorder**

**Additional methodological details**

*Participant instruction*

Between the ‘discovery’ and fMRI versions of the Go-NoGo task, participants were explicitly instructed through a computer program supervised by a trained research assistant. They were told, on a screen, ‘The best response for the stimulus [picture shown] is NOT TO PRESS so as to avoid loss’, followed by some practice trials of not-pressing. Then they were told ‘The best response for [next picture] is PRESS to win points’, followed by some practice trials until they practiced with all 4 stimuli. They then had to practice responding to all the stimuli in mixed order. If in this last practice session there was less than 90% success, the program alerted the research assistant to repeat the instruction procedure. This was very rarely necessary, as most participants had either worked out the correct answers anyway during the ‘discovery’ phase, or only needed to learn about the occasional stimulus (amongst the four) that they got wrong. Thus, essentially all subjects had the same number of practice trials.

**Group comparison methodology**

We tested for differences in a stepped manner, starting from the most agnostic and least prone to possible biases in constraining assumptions, to the most sensitive but somewhat more prone to bias. All methods were based on regressors of interest at the individual level, anchored either at cue onset (re. decision-making) or at outcome onset (re. sensitivity to outcomes and reward prediction errors) as described in the main text. At onset, we formed contrasts for:

1. the four main experimental conditions (Go-to-Win, Go-to-Avoid-Loss etc.; GtW, GtAL, NGtW, NGtAL)
2. Main effect of action, Go > NoGo := (GtW+GtAL)-(NGtW+NGtAL), and similarly for negative, Go < NoGo
3. Main effect of value, High expected value > Low expected value := (GtW+GtW)-(NGtW+NGtAL) and similarly for its opposite
4. Pavlovian-consistent > Pavlovian-inconsistent := (GtW+NGtAL) – (GtAL+NGtW) and again similarly for its opposite.

At outcome, we examined only trials where the correct response was performed, forming

1. Wins in GtW, null outcomes in GtW, Losses in GtAL, null outcomes in GtAL and similarly for the other two conditions.
2. ‘Win’, ‘Null’ and ‘Loss’, grouping the above.
3. Better-than-expected-Action events (bteGo for short), including ‘Win following correct Go-to-Win action’ and ‘Null following correct Go-to-Avoid-Loss action’.
4. Worse-than-expected-Action events (wteGo) included ‘Null following correct Go-to-Win action’ and ‘Loss following correct Go-to-Avoid-Loss action’.
5. Analogous groupings for better-than-expected following inaction (bteNoGo) and worse-than-expected following inaction (wteNoGo).
6. RPE regressors of ‘better than expected’, bte := bteGo + bteNoGo and ‘worse than expected’, wte := wteGo + wteNoGo . This follows the methodology of


   [1] – note that it controls for expected value.

Our most agnostic method used whole-brain analyses with the complete regressors of interest from the individual level. For example, to test whether areas that showed increased activation in response to Pavlovian-inconsistent, and in that sense unusual, stimuli, we performed a two-sample t-test using the second contrast 4. above. As another example, to test whether activation in response to the main effect of action varied with depression level, we covaried the first contrast 2. above with Hamilton scale scores.

Less agnostic and more powerful is the methodology that has been used to study pharmacological manipulation based groups in the Go-NoGo task


[2]. We used this to define functional ROIs. Here we note that in the present study, this method carries a small risk of bias. As there are more (33 vs. 20) participants with depression, the ROIs may be biased towards those areas that show significant contrasts in the depression group. In theory, it may exclude some voxels less activated in depression than in health, which would be of crucial interest in our study. In practice the greater power of this method identified clusters with similar peaks, but greater size, than clusters based on either group on its own, providing reassurance here.

Most constrained was the use of predefined, anatomical ROI masks. We used those mainly for those areas that we made specific hypotheses about but where the more data-driven methods above did not provide clear guidance. These were the Substantia Nigra / Ventral Tegmental Area, which is difficult to locate without masking, and the anatomical ventral striatum. We specifically used the latter to look for reward-prediction-error differences.

**Brain responses to reward prediction error**

In order to explore brain responses to reward prediction errors (RPE), and subsequently examine whether these differed in depressed participants, we used the regressors 6. and 10. above. We note, first of all, that in this study we did not manipulate the two components of reward prediction error, namely expectation and outcome, independently. Therefore our task is not optimized to identify brain regions whose activity correlates with outcome but is anticorrelated with expectation


[3]. However this is not relevant to our study question, which is about differences between healthy and control participants. Here, an RPE signal is represented in the brain, and this is different in depression vs. health, then if we can control for expectation, the group difference should be reflected in the response to outcome. As we have taken measures to control for the role of expectation, as detailed below, in the light of all the existing evidence for RPEs in the brain we can be reasonably confident that differential RPEs between the groups should be reflected at responses to outcome (controlling for action/inaction as well as for expectation).

We first examined the simple contrasts win-loss and loss-win (6.), as described in the main text. The areas sensitive to these outcome contrasts are illustrated in Figure A. We found no significant differences between the groups in the activation of these areas.

We also used the RPE regressors above (10.). These control for expected value as follows. Within each participant, the better-than-expected – worse-than-expected contrast is balanced for expectation (for the Q values), assuming that these Q values are stable and performance is near-optimal in all conditions, as above. Under such performance half of the ‘bte’ trials are high-Q and half low-Q, and exactly the same obtains for the ‘wte’ trials.

| **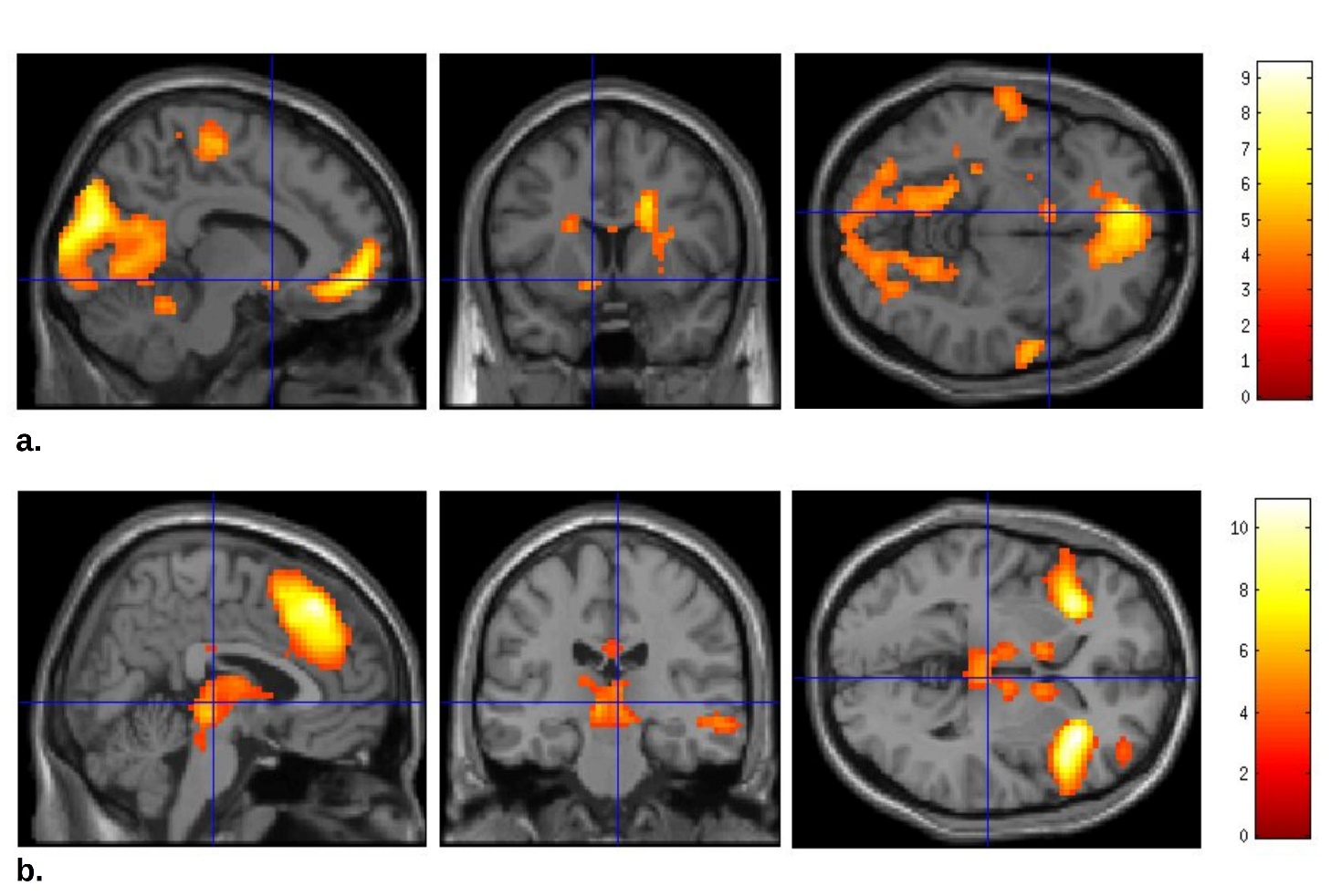**  **Figure A** |
| --- |

Pooled over all conditions and with FWE 5% significance level, the bte-wte contrast revealed significant clusters in many regions, including left medial prefrontal, bilateral superior visual areas and bilateral primary motor areas. Ventral striatal area responses could only be detected at the much more liberal *p*=0.005 uncorrected level. The wte-bte contrast revealed prominent FWE corrected clusters including bilateral anterior insulae, left premotor cortex and bilateral parietal cortices (all *p* _FWE_ < 0.001). Again at the 5% FWE level of significance we did not detect significant subcortical clusters. All these analyses are consistent with win-lose / lose-win contrasts above, with the exception of the tectal peak which was significant at 5% FWE only at the lose-win analysis. Again, none of the clusters identified showed a significant between-group difference. Here we extended the analysis using both anatomical (bilateral nucleus accumbens) and spherical predefined masks, but again we detected no group differences.

We then examined RPE by action interactions. The (bteGo – wteGo) – (bteNoGo – wteNoGo) contrast showed a small cluster significant at 5% FWE in the left mPFC, with coordinates MNI -3, 35, -20. For the opposite contrast, (wteGo – bteGo) – (wteNoGo – bteNoGo), a left anterior insula cluster survived (MNI -33, 26, -8). In an exploratory analysis, we then looked at the same contrasts at p=0.001 uncorrected and, for the purposes of exploratory group comparison, we defined functional ROIs based on the aforementioned mPFC and insular clusters. Figure B shows the average contrasts in these ROIs, demonstrating that following action (Go condition) there are stronger contrasts in both the mPFC area (sensitive to bte-wte) and in the insular area (sensitive to the opposite contrast).

| **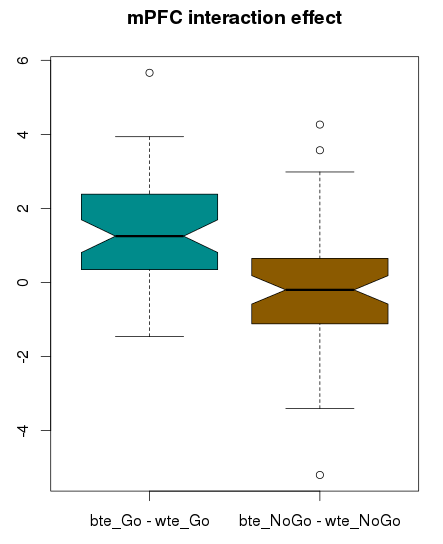 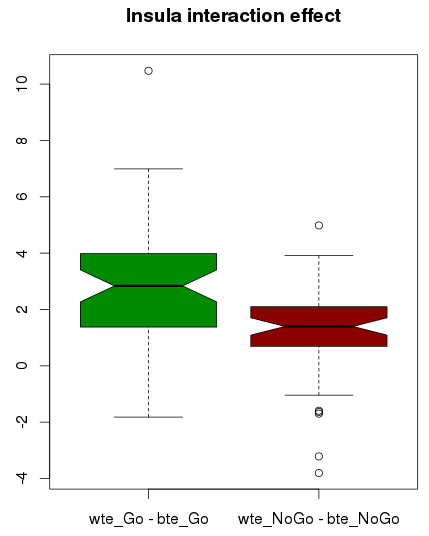**   1. **b.**   **Figure B** |
| --- |

**Exploratory findings in Supplementary Motor Area**

Here we describe an intriguing exploratory findings which merit attention in replication studies. The bilateral supplementary motor area (SMA) showed a clearly stronger activation during anticipation of action rather than inaction. The cluster significant at *p* _FWE_ < 0.01 is shown in Figure Ca. As the Bayesian analysis (table 1 in main text) showed some evidence against the null hypothesis of no difference between the groups, we examined the nature and direction of this difference. We found that the depressed group tended to have greater activation during action anticipation (Figure Cb, Cc ‘Action’) but there was no difference as a function of anticipating winning or losing, actual action performed or actual outcome received (Figure Cc). Furthermore, there was no correlation between this anticipatory activation and reaction time. If this finding is replicated, it would be interesting to see if it may be explained by the greater subjective experience that depressed participants have been reported to show


[4].

| **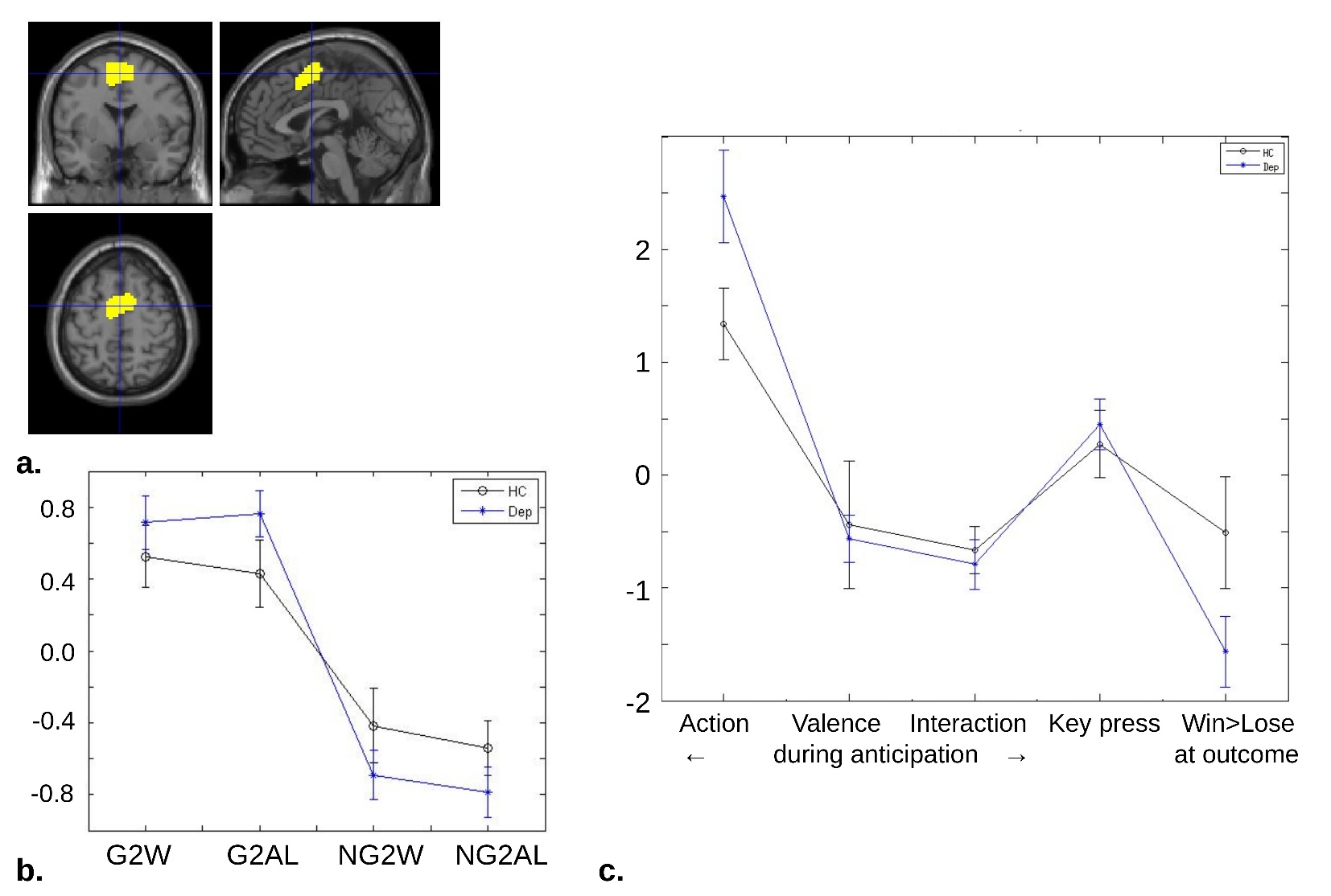**  **Figure C** |
| --- |

**References to online supplement**

1. Kurniawan IT, Guitart-Masip M, Dayan P, Dolan RJ. Effort and valuation in the brain: the effects of anticipation and execution. The Journal of Neuroscience. Soc Neuroscience; 2013;33: 6160–6169.

2. Guitart-Masip M, Economides M, Huys QJM, Frank MJ, Chowdhury R, Duzel E, et al. Differential, but not opponent, effects of L-DOPA and citalopram on action learning with reward and punishment. Psychopharmacology. Springer; 2014;231: 955–966.

3. Rutledge RB, Dean M, Caplin A, Glimcher PW. Testing the reward prediction error hypothesis with an axiomatic model. The Journal of Neuroscience. Soc Neuroscience; 2010;30: 13525–13536.

4. Clery-Melin M-L, Schmidt L, Lafargue G, Baup N, Fossati P, Pessiglione M. Why don’t you try harder? An investigation of effort production in major depression. PloS one. Public Library of Science; 2011;6: e23178.
