# Supplementary material for: Neural activity and fundamental learning, motivated by monetary loss and reward, are intact in mild to moderate major depressive disorder
Source: PLoS One. 2018 Aug 2;13(8):e0201451. doi: 10.1371/journal.pone.0201451 (PMC6072018; doi:10.1371/journal.pone.0201451)
Supplement: S2 File — This is a document detailing the variable names in the processed and raw data provided, and also how to access the imaging data uploaded into Neurovault. This document also explains the contents of the zip files HealthyControlBehavioralData.zip and DepressionGroupBehavioralData.zip which include the raw ‘discovery task’ data. (DOCX) [file pone.0201451.s002.docx]

**Instructions to using the data on which results in ‘Intact neural mechanisms subserving learning about monetary outcomes in moderate major depressive disorder’, by Moutoussis et al, are based**

General comments

In all files where participants are enumerated, numbers below 100 refer to healthy controls (in practice, less than 30) and numbers of 100 or over to participants suffering from Major Depressive Disorder (MDD).

**Self-report and behavioural data**

These are provided first, in the accompanying comma-separate-variable files, questionnaire_data.csv and ModelParameters.csv and secondly as raw behavioural data for the ‘Discovery task’ which served for model fitting and model comparison. This is described below.

The model parameters file contains the point estimate of each model parameter for each participant under the two statistical models that were compared, i.e. ‘all together’ or ‘separate group’ hierarchical Bayesian fits. The columns of the file are:

"pt_ID" Numerical ID

"bet_Appet.tog" Beta Appetitive, all participants fitted with common group prior, ‘together’ "bet_Aver.tog" Beta Aversive, ‘together’

… [See main text for list of parameters]

“iLik.tog" Integrated likelihood, ‘together’

"bet_Appet.sep" Beta Appetitive, ‘separate’ group fit

…

"iLik.sep" Integrated likelihood, ‘separate fit’

The questionnaire spreadsheet file is mostly self-explanatory, containing in the first few columns a numerical ID, experimental group, age, gender, years in full time education (schyear), mediations, whether hospitalized for a medical condition, medicinal drugs, recreational drugs, etc. as per table 1 of the main text. Then, in columns 11-100 one finds the item-by-item data of the mood related questionnaires, i.e. in order the PHQ, HAM-D, SHAPS, and BDI. The total scores of the first three are also provided for convenience, as ‘phqtotra’, ‘hamtotra’ and ‘sscore’.

Raw data for the ‘Discovery’ version of the task are provide in zip files DepressionGroupBehavioralTask.zip and HealthyControlBehavioralTask.zip . Each consists of the full set of individual data, each comprising a matlab data file [ID]*LearnVerDat*[TIMESTAMP].mat containing an array. The colums of the array are as follows.

1. count of the trial, 1 to total

2. Trial stimulus, e.g. 2-> stimulus used for GoToAvoidLoss, 3->NoGoToWin

3. Time of stimulus onset

4. (not used)

5. Time interval from stimulus to 'target', i.e. signal that decision must now be implemented.

6. (not used)

7. (not used)

8. Index as to whether the 'target' will appear at all (for skipping targets during the scanner task only)

9. (not used)

10. Response key, i.e. code of key pressed eg 71=SPACEBAR, 0 if none

11. Time of key press

12. RT, reaction time

13. Response, Go or NoGo etc.

14. TargetDispT interval that target was displayed for

15. Time when Outcome was displayed

16. Inter Trial Interval (jitter)

17. Play-money cents won.

**Scan data**

These include the second-level contrast files that arise from the 2 x 2 ANOVAs described in the text. The key contrast has as factor 1 the action and factor 2 the valence at the point of stimulus presentation, that is, when participants form anticipations or predictions of the outcomes. Users can use the ‘Results’ section of the SPM software to load the SPM.mat files provided in Neurovault. Here is how to access them, for example by searching for the term ‘Go-NoGo’ in the main of Neurovault:

| 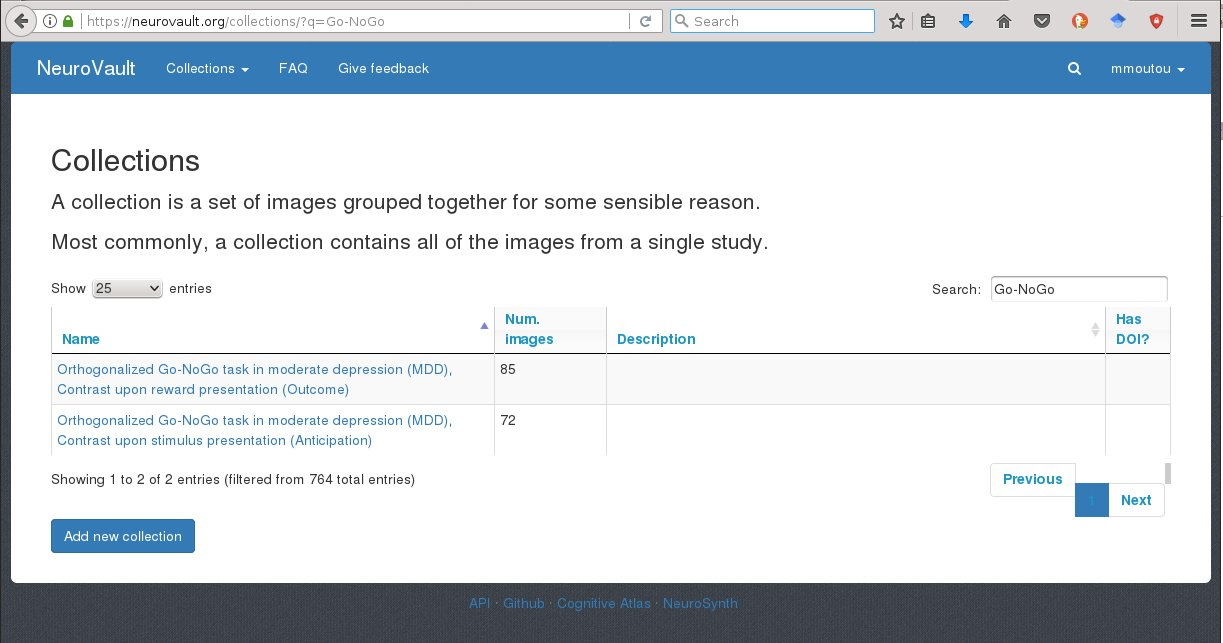 |
| --- |

The corresponding first-level contrasts in the Anticipation files are arranged as Action x Valence, as follows. [1 0 0 0] : GoToWin ; [0 1 0 0] : Go to Avoid losing; etc. for the four conditions. Hence, inference [1 -1 1 -1] tests for the main effect of Value, i.e. Win-Loss conditions at anticipation (which is, actually, unremarkable). The SPM.mat files already include the key contrasts described in the text.

The SPM.mat file at Outcome is arranged as Action x Prediction-Error, so [1 -1 1 -1] means ‘Better-than-expected minus Worse-than-expected summed over both Action emission and withholding’ . This produces maps like the following one, which is thresholded at the FWE 0.05 level. This shows significance maps and also the contrast and design matrix. From here the user can reproduce the selection of functional maps.

| **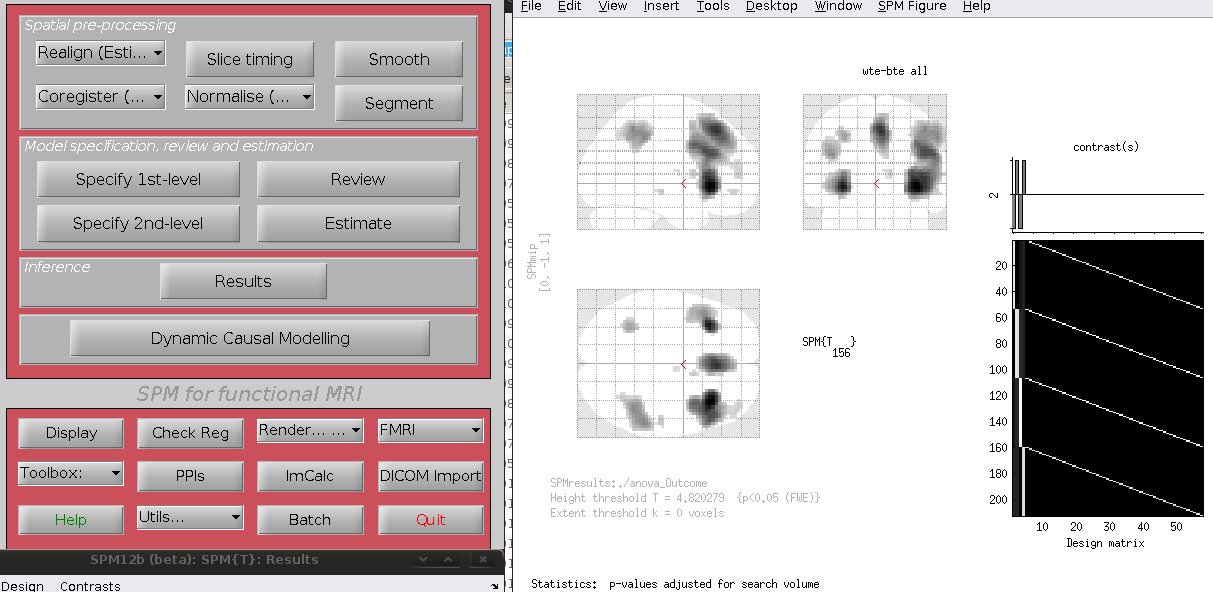** |
| --- |
